# High-speed high-resolution data collection on a 200 keV cryo-TEM

**DOI:** 10.1107/S2052252522000069

**Published:** 2022-01-29

**Authors:** Jared V. Peck, Jonathan F. Fay, Joshua D. Strauss

**Affiliations:** a Biochemistry and Biophysics, University of North Carolina at Chapel Hill, 101 Mason Farm, Chapel Hill, NC 27599, USA; b Biochemistry and Biophysics, 6107 Thurston Bowles Building, Chapel Hill, NC 27599, USA

**Keywords:** cryo-electron microscopy, 200 keV cryo-TEM, beam image shift, direct-electron detector

## Abstract

Limitations to successful single-particle cryo-electron microscopy (cryo-EM) projects include stable sample generation, production of quality cryo-EM grids with randomly oriented particles embedded in thin vitreous ice and access to microscope time. To address the limitation of microscope time, methodologies to more efficiently collect data on a 200 keV Talos Arctica cryo-transmission electron microscope at speeds as fast as 720 movies per hour (∼17 000 per day) were tested. In this study, key parameters were explored to increase data collection speed including: (1) using the beam-image shift method to acquire multiple images per stage position, (2) employing UltrAufoil TEM grids with R0.6/1 hole spacing, (3) collecting hardware-binned data and (4) adjusting the image shift delay factor in *SerialEM*. Here, eight EM maps of mouse apoferritin at 1.8–1.9 Å resolution were obtained in the analysis with data collection times for each dataset ranging from 56 min to 2 h. An EM map of mouse apoferritin at 1.78 Å was obtained from an overnight data collection at a speed of 500 movies per hour and subgroup analysis performed, with no significant variation observed in data quality by image shift distance and image shift delay. The findings and operating procedures detailed herein allow for rapid turnover of single-particle cryo-EM structure determination.

## Abstract 2

This study explores parameters for high-speed, high-resolution, single-particle data collection using a 200 keV Talos Arctica equipped with a Gatan K3 DED. Near-atomic resolution electron microscopy maps were obtained from datasets collected as fast as 720 movies per hour.

## 1. Introduction

Single-particle cryo-electron microscopy (cryo-EM) is becoming the predominant structural biology technique for obtaining 3D structures of purified macromolecular complexes. Structural determination of novel protein complexes with *C*1 symmetry at 2.5–4 Å resolution using a cryo-transmission electron microscope (cryo-TEM) with autoloader and direct-electron detector (DED) is now routine (Cheng, 2015 ▸). The choice of cryo-TEM model, camera and operational voltage can vary; however, a 300 keV cryo-TEM with 3 or 4 condenser lenses is ideal for high-resolution data collection. Structures of test samples such as apoferritin and adeno-associated virus have been determined with different types of high-end, 300 keV cryo-TEMs at atomic to near-atomic resolution 1.13–1.56 Å (Nakane *et al.*, 2020 ▸; Yip *et al.*, 2020 ▸; Xie *et al.*, 2020 ▸). Advantages of 300 keV cryo-TEMs include higher optical resolution and less image distortion including Ewald sphere and phase errors due to beam-tilt induced coma (Zhu *et al.*, 2018 ▸; Glaeser *et al.*, 2011 ▸; Zhang & Zhou, 2011 ▸). However, for biological samples the optical resolution of the transmission electron microscope (TEM) is not the main limiting factor for cryo-EM, rather it is the sample and ice thickness (Kim *et al.*, 2018 ▸). As the depth of field and mean free path of electrons in vitreous ice increase with energy, 300 keV cryo-TEM can facilitate imaging at higher maximum resolution for thicker specimens compared with the 200 and 100 keV counterparts (Grimm *et al.*, 1998 ▸; Zhang & Zhou, 2011 ▸).

Mid-level 200 keV cryo-TEMs equipped with DEDs are also capable of obtaining near-atomic resolution of protein complexes ranging in size from several MDa to as small as 64 kDa (Campbell *et al.*, 2014 ▸; Herzik *et al.*, 2017 ▸, 2019 ▸; Hamdi *et al.*, 2020 ▸; Danev *et al.*, 2021 ▸; Cao *et al.*, 2021 ▸). Two test samples, mouse apoferritin at 1.75 Å and beta-galactosidase at 1.8 Å resolution, have been determined using a 200 keV cryo-TEM in super-resolution mode (Merk *et al.*, 2020 ▸; Wu *et al.*, 2020 ▸). As of September 2021, there are 297 EM maps deposited in the Electron Microscopy Data Bank (EMDB), collected with a Talos Arctica which are <4 Å resolution (Patwardhan, 2017 ▸). Since 200 keV cryo-TEMs cost significantly less than their 300 keV counterparts and are also capable of obtaining EM maps at near-atomic resolutions, we aim to further expand their utility as high-resolution data collection instruments (Mills, 2021 ▸; Sader *et al.*, 2020 ▸). Importantly, the methods for acceleration of data collection described herein will also aid in more rapid data collection for other 200 and 300 keV cryo-TEMs equipped with DEDs. As the demand for access to cryo-TEMs capable of obtaining near-atomic resolution maps increases, making efficient use of the available microscope time for high-resolution data collection is critical for the success of cryo-EM laboratories, multiuser core facilities and for individual researchers. In this study, we focus our discussion on improving high-resolution data collection rates for a 200 keV Talos Arctica equipped with a Gatan K3 DED.

Several approaches have been used to increase throughput for single-particle cryo-EM data collection and are often dependent on the microscope setup, hardware, detector and image acquisition software. One way to maximize beam-time efficiency is by increasing particle number per micrograph by lowering the magnification, thereby increasing the field of view of the detector. This is often accomplished with a high-speed counting DED such as a Falcon 4, or a Gatan K2 or K3 camera operated in super-resolution mode, which can effectively quadruple the number of image pixels at a given magnification (Sun *et al.*, 2021 ▸; Li *et al.*, 2013 ▸; Chiu *et al.*, 2015 ▸; Guo *et al.*, 2020 ▸). This approach has been utilized to determine near-atomic models at greater than physical Nyquist of the detector for symmetric protein complexes, even from a single micrograph (Feathers *et al.*, 2021 ▸). Operating a Falcon 3EC or DED64 detector in integrated mode rather than counting mode can also increase data collection rates. Integrated mode is faster than counting mode for these DEDs (Mendez *et al.*, 2019 ▸; Song *et al.*, 2019 ▸), but faster data collection in integrated mode typically results in lower resolution in the final EM maps. Thus, the choice of camera and parameters used for data acquisition are critical in the optimization of data collection speed and EM map quality.

Another common method to increase data acquisition rates is to collect multiple micrographs from a single stage position by shifting the beam hole to hole and acquiring a single micrograph at each hole. This beam-image shift (BIS) method is standard practice for automated single-particle cryo-EM data collection (Cheng *et al.*, 2018 ▸). Since each stage movement induces mechanical vibrations and residual stage drift, potentially degrading image quality, a delay after each stage movement is often included in automated data collection. It is therefore advantageous to collect as many micrographs as possible per stage position to reduce the overall time spent on stage movement and waiting for the stage to settle, thereby increasing the efficiency of data collection. One detriment of the BIS method is that image shifts lead to off-axis coma and introduce higher-order aberrations that can degrade micrograph quality and limit the resolution of EM maps (Zhang & Zhou, 2011 ▸; Glaeser *et al.*, 2011 ▸). The off-axis coma can be compensated for during data collection by imposing beam tilt in the direction opposite to the image shift and is now standard in multiple data collection software programs including *EPU*, *Latitude*, *Leginon* and *SerialEM* (Cheng *et al.*, 2021 ▸; Schorb *et al.*, 2019 ▸). In addition, higher-order aberrations caused by BIS can be corrected in *RELION* and *cryoSPARC* (Punjani *et al.*, 2020 ▸; Zivanov *et al.*, 2018 ▸).

When using the BIS method for data collection, a delay is applied after each BIS to allow lens hysterics, stigmators and the electron beam to stabilize, hereafter referred to as image shift delay. In *SerialEM*, the image shift delay in seconds is calculated independently for each applied BIS and is typically linear to the distance of the shift in micrometres, such that a 1 µm shift applies a 1 s delay before acquiring an image by default. The optimum image shift delay for a given microscope can be calibrated and is stored in the *SerialEM* properties file as a table. The imposed image shift delay can be changed in the *SerialEM* interface by modifying the image shift delay factor, which is set to 1 by default and can be scaled up or down. The amount of delay required for a given image shift distance can vary between instruments. As described in the *SerialEM* online documentation, the image shift delay is particularly important for FEI microscopes, but the long delays induced by large BIS can be excessive (Mastronarde, 2021 ▸). Thus, reducing the image shift delay has significant potential to increase data collection speeds. Setting the image shift delay factor to 0 has been shown to increase data collection rates using a JEOL CRYO ARM 300 without compromising data quality when collecting BIS up to 7.5 µm to obtain a 1.7 Å structure of apoferritin, achieving rates of 250–375 movies per hour (Efremov & Stroobants, 2021 ▸). However, the optimal image shift delay for a Talos Arctica and the effect that reducing this delay has on EM map quality when using the BIS method for data collection has not been experimentally determined.

The use of the BIS method in automated data acquisition has provided significant increases in data collection speeds (Bromberg *et al.*, 2020 ▸; Cash *et al.*, 2020 ▸; Cheng *et al.*, 2018 ▸; Wu *et al.*, 2019 ▸). To take further advantage of this technique, it is also possible to obtain multiple images within a single Quantifoil hole, though this approach is restricted to high-end cryo-TEM with three condenser lenses, such as a Titan Krios, or the four-condenser lens JEOL CRYO ARM 300, which can expand or contract the diameter of the electron beam while maintaining parallel illumination. When used with a 300 keV cryo-TEM capable of fringe-free illumination, it is possible to collect as many as 10 images in a single 2 µm-diameter Quantifoil hole (Weis & Hagen, 2020 ▸). This is not feasible when using two-condenser-lens optical systems, which require adjustments to the strength of the C2 lenses to obtain parallel illumination (Herzik *et al.*, 2017 ▸; Herzik, 2021 ▸). Microscopes with two-condenser-lens systems including a ThermoFisher Scientific Talos Arctica and Glacios are limited to obtaining a single micrograph for most Quantifoil TEM grids (with the hole size between 0.6 and 2 µm) when using a 50 µm condenser aperture to limit beam diameter, see Table S1 of the supporting information.

We explore different *SerialEM* and DED camera parameters for single-particle cryo-EM data collection, using a 200 keV ThermoFisher Scientific G3 Talos Arctica equipped with a Gatan K3 DED. We focus on increasing the rate of data collection by the BIS method, collecting one image per hole and using various settings in *SerialEM*. Beam-tilt compensation, as implemented in *SerialEM* (coma versus image shift), was utilized to correct for off-axis coma induced by shifting the beam away from the optical axis. By combining large BIS up to ∼7 µm with a reduced image shift delay, we obtained a data collection speed of approximately 520 movies per hour. We attained faster data collection speeds when collecting hardware-binned movies compared with collecting in super-resolution mode, with super-resolution and hardware-binned maps having similar resolution. We report a collection rate of up to 524 movies per hour without compromising data quality and present a 1.78 Å resolution map of mouse apoferritin that was obtained from a single 11.7 h data collection session of 5881 micrographs. Moreover, we propose that a cryogrid with a hole spacing of R0.6/0.5 can allow for data collection speeds of more than 720 movies per hour using a shorter BIS distance (∼6.2 µm) and image shift delay factor settings utilized for data presented herein, with the potential to increase data collection speeds further by using larger BIS distances.

## 2. Methods

### 2.1. Sample preparation for cryo-EM

Expression plasmid encoding mouse heavy chain apoferritin, pET24a-mFth1, was kindly provided by Dr Masahide Kikkawa of Tokyo University School of Medicine. Protein was expressed in *E. coli* BL21(DE3)pLysS cells and purified using published protocol. Aliquots of purified apoferritin at 24.81 mg ml ^−1^ in buffer A (30 m*M* HEPES pH 7.5 150 m*M* NaCl, 1 m*M* DTT and 5% trehalose) were flash-frozen in liquid nitro­gen and stored at −70°C for later use. Frozen aliquots were thawed at 37°C or on ice and stored at 4°C for no more than 1 month. The apoferritin stock solution was diluted to the final concentration of 5 mg ml ^–1^ in buffer B (30 m*M* HEPES, 150 m*M* NaCl, 1 m*M* DDT, pH 7.5). UltrAufoil TEM grids were plasma cleaned with a TergeoEM (Pie Scientific) at 15 W for 60 s with direct plasma using a 25:75 ratio of oxygen to argon. Cryogrids were then prepared using a ThermoFisher Vitrobot MK IV by rapid immersion in liquid ethane propane (40:60 mixture) cooled to approximately −186°C. The sample was applied directly to the surface of an R0.6/1 300 mesh UltrAufoil TEM grid and blotted with Whatman 595 filter paper for 4 s at 95% humidity and 25°C using a blot force of −10.

### 2.2. Cryo-EM data collection setup

**Figure 1:**
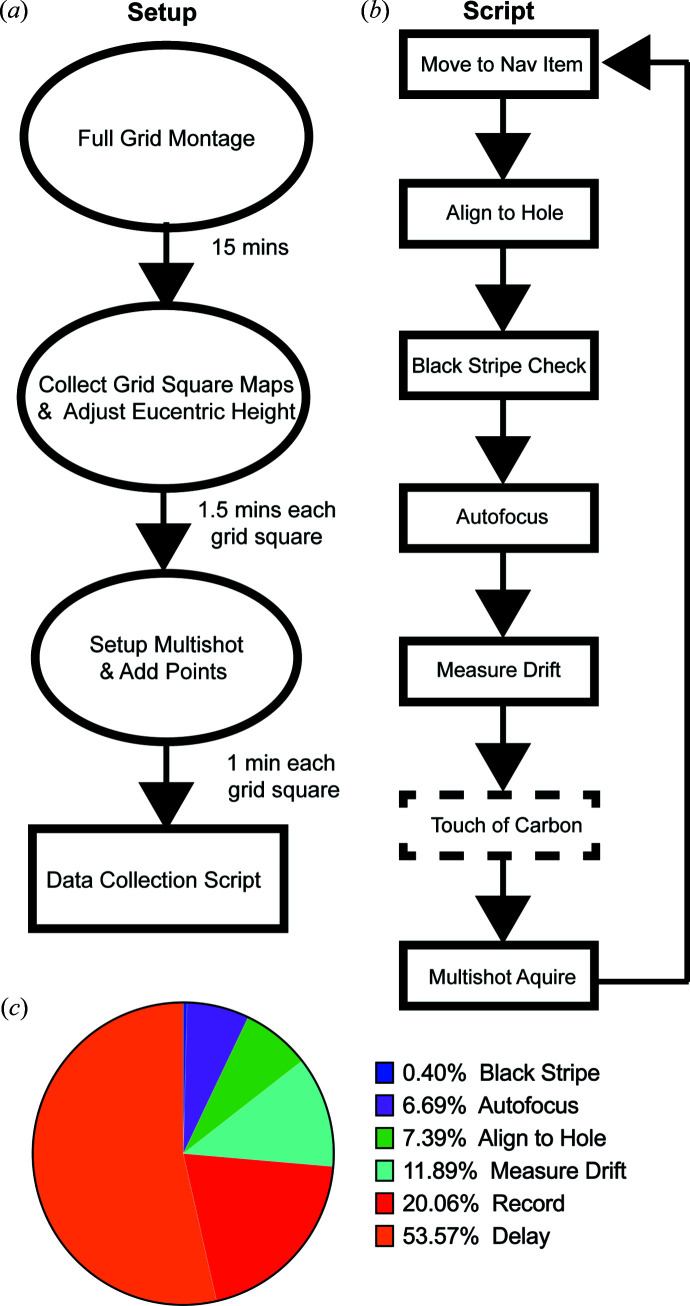
Data collection setup and *SerialEM* script. (*a*) Flowchart outlining steps and the time to set up data collection. A full-grid montage is collected at 62× magnification in lower magnification (LM) mode to identify areas ideal for data collection. Maps of selected grid squares are acquired at 210× magnification in LM mode after finding eucentric height. Multishot record parameters and points are added to each of the grid square maps by microscope operator. (*b*) Flowchart of the *SerialEM* script used to collect data. The stage is moved to each of the points (in *XYZ*) and centered over the hole iteratively using the reference image. Errors in the camera sensor referred to as black strip are checked after hole centering. Autofocus using beam-tilt iterates until target defocus is obtained. Stage drift is measured 20 times, or until drift threshold is obtained. As an option, an additional beam shift is applied to direct the beam away from the center of the hole, we refer to this as touch of carbon and find this useful for particles that cluster close to the edge of the carbon film. Micrographs are acquired in a multishot array, one micrograph is taken at each Quantifoil hole, typically 9–49 holes depending on the hole spacing. (*c*) Percentage of time spent on each step of the data collection script. Record and delay occur during multishot acquire, with delay including image shift delay, other *SerialEM* delays (ExtraBeamTime, ShutterDeadTime, ExtraOpenShutterTime), and camera read out and file write time.

All cryo-EM data analyzed in the study were collected on a 200 keV ThermoFisher Scientific G3 Talos Arctica equipped with a Gatan K3 DED. The microscope was aligned using a cross-grating replica 2160 mm (TedPella) TEM grid, this took approximately 10–15 min to complete. The pixel size was calibrated by comparing apoferritin maps with that of the known atomic structure (PDB entry 6v21; Wu *et al.*, 2020 ▸) in chimera (USDS) (Pettersen *et al.*, 2004 ▸). Parallel illumination was obtained by adjusting the C2 lens in diffraction mode at 850 mm (Herzik, 2021 ▸). Using a 50 µm condenser aperture limits the beam diameter to 1.6 µm under parallel illumination, which allows for the acquisition of one micrograph per hole on a Quantifoil TEM grid (Table S1). Coma-free alignment was achieved in *SerialEM* and verified by acquiring 3 × 3 Zemlin Tableau (Zemlin *et al.*, 1978 ▸). Coma versus image shift calibration was done in low-dose mode using a 7 µm BIS in the *X* and *Y* directions. Data were collected in a semiautomated fashion using *SerialEM* (Mastronarde, 2005 ▸). Movies were recorded as LZW compressed TIFFs and gain-corrected in *cryoSPARC Live* (Punjani *et al.*, 2017 ▸). The 50 µm condenser aperture and 100 µm objective aperture were inserted during data collection. The data collection script was made by modifying scripts written by Wim Hagen (SPA multiple images, test hole centering and k3blackstripecheck) and Chen Xu (WaitingForRefilling), which are available in the *SerialEM* script depository. The workflow of automated data collection can vary by personal preference, software and hardware differences in each microscope, but the steps detailed here represent the foundations retained for most microscope setups (Fig. 1 ▸). Automated data collection proceeds through a custom *SerialEM* script, which takes ∼1–2 h to set up a normal overnight data collection, with the most time spent montaging the grid and marking areas to collect. To address the issue of imaging particles that localize at the edge of the hole in very thin ice, we will occasionally include an extra BIS (100–300 nm) away from the center of the hole, which is applied before taking a multishot, we refer to this option as a ‘touch of carbon’. Our data collection script also includes commands which output the amount of time spent in each step of the data collection process to the log file, which we then use for further analysis. A gain reference was collected in *Digital Micrograph* (Gatan) before starting data collection which took ∼15 min to collect. A new dark reference was collected every hour during data collection, as implemented in the *SerialEM* data collection script. The speed of data collection was determined by reading the timestamps of the raw movies. All micrographs were processed in *cryoSPARC* (version 3.2.0) using octahedral symmetry for 3D refinements (see below).

### 2.3. Single-particle cryo-EM data collection and image processing with different image shift delay factors

To compare the effects of changing the image shift delay on data quality, we collected 8 datasets consisting of 490 movies each from a single UltrAufoil cryogrid prepared as described above. Data were collected using nominal magnification of 54 900× corresponding to a pixel size of 0.88 Å, an exposure time of 2.716 s and a total dose of 45.4 e^−^ Å^−2^. All areas selected for data collection were judged to be of similar ice thickness and particle density. Grid squares were selected based on appearance at low magnification, and screening the grid identified areas of thin ice with mostly a single monolayer of apoferritin. Each dataset consisted of ten 7 × 7 multishots collected on two adjacent grid squares to ensure similar total stage movement distance and time between groups. Data were collected in either super-resolution mode or binned by a factor of 2 in hardware, hereafter referred to as ‘hardware-binned’. Five datasets were collected in super-resolution mode with image shift delay factors of 0, 0.25, 0.5, 1 and 2. Three hardware-binned datasets were collected with image shift delay factors of 0, 0.5 and 1.

All datasets were processed on the same workstation using *cryoSPARC Live* with a consistent workflow, as detailed below. All eight datasets were imported into *cryoSPARC Live* during data collection using a calibrated pixel size of 0.88 Å for the three hardware-binned datasets and 0.44 Å for the five super-resolution datasets. Micrographs were not curated based on CTF resolution cut-off, rather all movies were included in the analysis, and junk particles were removed after template picking and selection of 2D classes with clear secondary structure. A total of 50 micrographs were used to generate a set of 2D class averages with visible secondary structure that were used as inputs for the Template particle picker. Template-extracted particles were subjected to 2D classification and any well centered classes that had clear secondary structure were carried forward for further analysis. An *ab initio* volume was generated from a subset of 40 000 particles for each of the datasets. Homogeneous refinement was carried out with all particles from the selected 2D classes using standard input parameters. Local CTF refinement, global CTF refinement and another round of homogenous refinement were carried out for each dataset. The centered particles were then re-extracted at a box size of 300 pixels for the hardware-binned data and 600 pixels for the super-resolution data to standardize the particle size to box size ratio between all datasets. The dataset collected with an image shift delay factor of 2 in super-resolution mode contained the fewest extracted particles at 313 040. To remove variation in particle number as a factor in the final EM map resolution, the particle sets tool job was utilized to randomly pick a subset of 313 040 particles from each dataset. The re-extracted particles were then imported into their respective homogenous refinement jobs, this time using the same *ab initio* volume as input for every refinement. Homogenous refinements were run, then a single iteration each of local CTF refinement, global CTF refinement and a final round of homogenous refinement. The final resolution and *B*-factor for each dataset are shown in Table 1 ▸.

### 2.4. Single-particle cryo-EM data collection and processing – overnight data collection

To acquire enough data to perform subgroup analysis and determine how reducing the image shift delay affected data quality using BIS up to 7 µm, we collected a single overnight dataset of apoferritin using an image shift delay factor of 0.5. The 1.78 Å resolution EM map of mouse apoferritin was generated from 5881 micrographs that were collected on an R0.6/1 UltrAufoil grid in 11.7 h using a 7 × 7 multishot. The images were collected in hardware-binned mode using a 0.5 image shift delay factor and a nominal magnification of 88 400×, equating to a pixel size of 0.545 Å at the detector level. The exposure time was 1.438 s, using 60 frames and a total exposure dose of 54.3 e^−^ Å^−2^. Raw movies were imported at a pixel size of 0.545 Å and gain corrected. Particles were picked with the template picker using references from a 2D classification of a 500 micrograph, blob-picked subset. Extracted particles (2.75 million, 440 final box size) were curated through multiple rounds of 2D classification, resulting in 1.1 million particles included in the first homogenous refinement. The particles were then split by micrograph into separate exposure groups, subsequent local CTF refinement, global CTF refinement and a homogenous refinement brought the resolution to 1.78 Å, *B*-factor 57.2. Further refinement resulted in a negligible improvement to 1.77 Å, *B*-factor 55.4. At this point, particles were split by distance from optical axis into ten groups with the multishot center position containing the fewest at 23 445 particles. To normalize particle number between groups, a random subset of 23 445 particles was selected from each group and individual homogenous refinements conducted. The group furthest from the optical axis, consisting of the four corners of the multishot, was further separated into two separate subgroups by the image shift delay (either 0.8 or 3.4 s). The first position requires application of a large BIS from the center of the multishot array to the corner and therefore induces a longer delay. The other three corners are approximately equidistant from the optical axis, but require shorter image shift delays. The long-delay group was the smallest and contained 20 564 particles, so a random subset of 20 564 particles was selected from the short-delay subgroup and individual homogenous refinements were conducted for each subgroup. Local resolution was calculated using a GPU implementation of the local windowed FSC method in the *cryoSPARC* software package using default input parameters (Cardone *et al.*, 2013 ▸).

### 2.5. High-speed cryo-EM data collection on a lacey carbon TEM grid

Movies were collected using a regular 9 × 9 multishot array, with 1.1 µm lateral spacing. Data were collected at an exposure of 0.905 s in 20 subframes to give a total exposure of 16.2 e^−^ Å^−2^. This resulted in 1134 images from 14 multishot positions on one 200 mesh lacey carbon grid square. A 24 micrograph subset of the selected 2D classified blob- and template-picked particles were manually inspected and used as a training set for the *Topaz* particle-picking software (Bepler *et al.*, 2020 ▸). After removal of duplicate particles, a single round of both 2D classification and multireference refinement resolved the final stack of 219 350 particles, creating a map with a nominal resolution of 2.19 Å after local CTF refinement and post-processing in *cryoSPARC*.

## 3. Results

### 3.1. Modulating the image shift delay factor to increase SPA data collection speed

**Figure 2:**
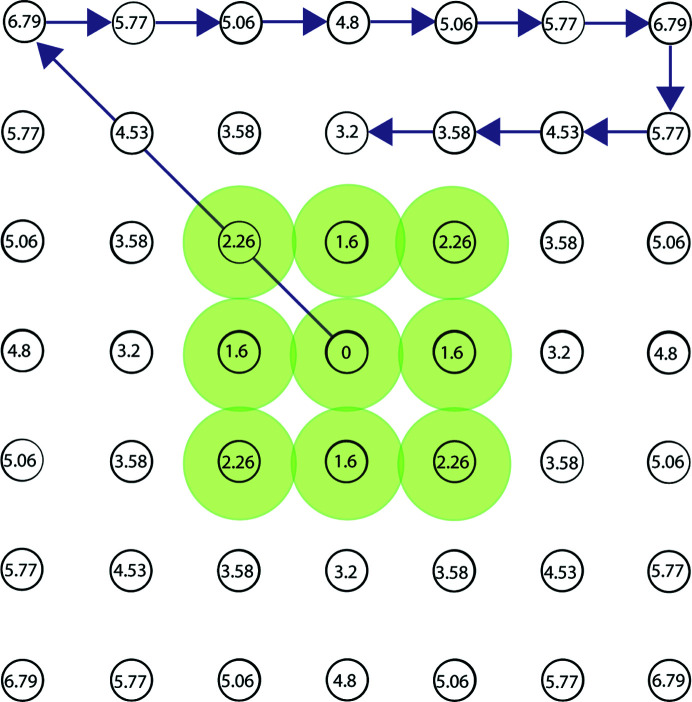
Multishot setup for Talos Arctica on an R0.6/1 Quantifoil TEM grid. The carbon foil hole and beam diameter (0.6 µm). (*a*) Holes and beam diameter (indicated by green circles) drawn to scale. The distance in micrometers from the center hole to adjacent holes is indicated at each position in the multishot. Direction and order of BIS are indicated by arrows.

In our analysis of data collection using an image shift delay factor of 1 and hardware-binning, we found that the majority of the time is spent in the ‘multiple record’ step. This includes the time taken to shift the beam, the image shift delay applied, other standard delays for the shutter, the exposure time, and the camera read out and file write time. After separating the exposure time for each record, we observed that more than 50% of total data collection time is spent in delay and read/write time [Fig. 1 ▸(*c*)]. The image shift delay is calculated after each beam-image shift and is proportional to the distance from the previous position, which can then be scaled by changing the image shift delay factor. Collecting data by BIS, as implemented in *SerialEM*, the beam shifts to a corner of the multishot array and then to adjacent holes, which means that the first image shift delay is the longest, with all other delays being shorter (Fig. 2 ▸). We sought to minimize delay time to improve data collection speeds by reducing the image shift delay and analyzing subsequent data quality for degradation.

**Figure 3:**
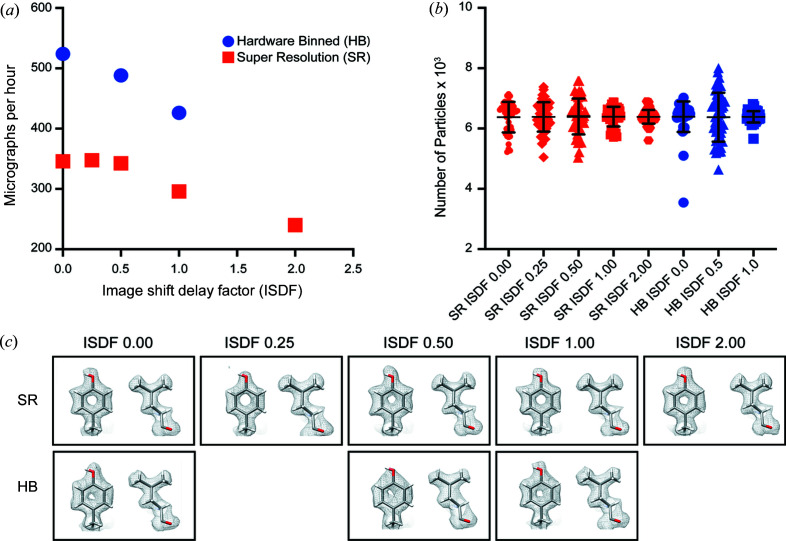
Data collection speeds using different image shift delay factors. (*a*) Rate of data collection using image shift delay factors of 0, 0.25, 0.5, 1 and 2 in super-resolution (SR) mode or hardware-binned (HB) mode. (*b*) Particle contribution of individual multishot positions contributing to the final 3D reconstruction for each of the recording conditions and image shift delay factor tested. The outlier in the hardware-binned image shift delay factor 0 is attributed to beam edge entering the field of the detector during data collection. For further breakdown of particle contributions see Fig S1. (*c*) Individual panels show visualization of coulombic potentials for amino acids Y29 and L115 for each of the eight apoferritin maps.

At a magnification corresponding to a calibrated pixel size of 0.88 Å, we found reducing the image shift delay factor could improve data collections speeds, with super-resolution mode consistently slower than hardware-binned mode [Fig. 3 ▸(*a*)]. To determine if large BIS distances at different speeds affect data quality, we counted the number of particles extracted from each multishot position that contributed to the final EM maps from each image shift delay factor and data collection setting. We found that each multishot position contributed a similar number of particles to the final reconstruction, indicating that the data quality showed no significant degradation when the image shift delay was reduced (Figs. 3 ▸ and S1 of the supporting information). This experiment suggests it is possible to obtain a 1.8 Å resolution EM map at speeds of up to 520 movies per hour using ∼7 µm BIS, reduced image shift delay factor and hardware-binning of data (Table 1 ▸).

**Figure 4:**
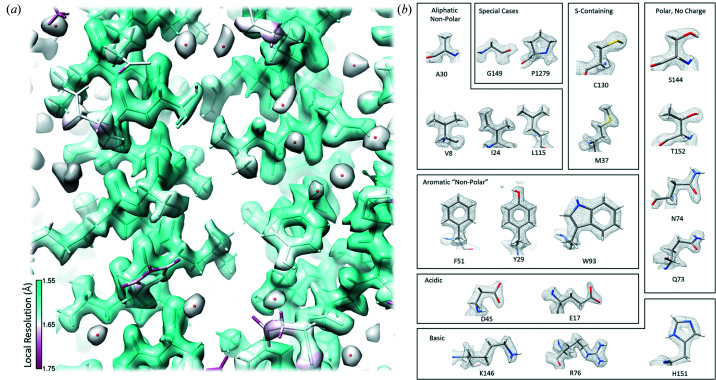
EM map of apoferritin collected overnight. (*a*) Local-resolution heat map for a region of the 1.78 Å overnight apoferritin reconstruction. Density contribution from water molecules is visible (red balls within gray transparent map). (*b*) Individual panels show the visualization of coulombic potentials for representative examples of each of the 20 amino acids. These data were collected with hardware-binning, using an image shift delay factor of 0.5 and collected at a rate of 500 movies per hour.

To further explore how reducing image shift delay affected data quality, we collected a larger dataset using an image shift delay factor of 0.5 and hardware-binned the movies. A 5881 movie dataset was collected overnight on a different mouse apoferritin UltrAufoil grid using higher magnification corresponding to a pixel size of 0.545 Å at the detector level and a shorter exposure time of 1.438 s. The data collection rate was measured at 500 movies per hour. The resolution of this map was 1.78 Å and contained high-resolution features expected for a sub-2 Å map, including distinctiveness of carbonyl, methyl and hydroxyl groups; holes in aromatic amino acid residues; and density of water molecules, illustrated in Fig. 4 ▸.

To determine the effect large BIS with reduced image shift delays had on data quality, we performed subgroup analysis of the particles that contributed to the final EM map by dividing them into groups by multishot position, see Table 2 ▸ and Fig. S2. During data collection the beam was shifted ∼7 µm to the corner of the multishot, then shifted by 1.6 µm laterally to adjacent holes. All multishot positions had a similar number of particles that contributed to the final EM map, though we noticed position 1 (largest BIS and the first micrograph taken) had 10% fewer particles than the mean contribution. We suspect the reduction in particles was caused by residual stage instability or insufficient image shift delay, as this was the first micrograph captured and the only position with a large BIS. To investigate how shifting the beam affected EM map quality, EM maps were reconstructed from ten BIS distance groups and two image shift delay groups with equal BIS distance, see Fig. S2. The resolution and *B*-factor were similar in each of the EM maps, all of which were 2.0 Å with a *B*-factor of 40–43 (Table 2 ▸). Reduced image shift delays did not degrade the EM map quality; the final resolution, *B*-factor values and high-resolution features present in each map were of the quality expected at 2 Å resolution.

### 3.2. Exploring parameters for faster data collection

Finally, we reasoned that even faster data collection could be achieved by reducing the exposure time and by collecting on a cryo-grid with an R0.6/0.5 hole spacing, which would allow for more images to be acquired at each stage position with a similar maximum BIS distance. To emulate this condition, we used a lacey carbon TEM grid and set the image shift delay factor to 0. We did not expect either the image shift delay factor of 0 or BIS up to 6.2 µm to adversely affect data quality based on our previous analysis. No longer confined to traditional hole spacing, we were able to set up a 9 × 9 multishot with a 6.2 µm maximum BIS on a lacey carbon grid and achieve a data collection speed of 720 micrographs per hour (Fig. S3). The exposure time was reduced to 0.9 s to provide faster data acquisition time. The data collection script was modified by removing the hole-centering step and the autofocus was conducted on the optical axis. Data collected from a single grid square achieved a nominal resolution of 2.2 Å (Fig. S3 and Table 1 ▸). We expect the resolution of the final EM map to be higher when collected on an UltrAufoil TEM grid compared with a Lacey carbon TEM grid, as we are typically limited to 2.0–2.2 Å resolution map reconstructions on carbon Quantifoil grids (data not shown), as others have reported (Russo & Passmore, 2014 ▸). This demonstrates that using a multishot array designed for an R0.6/0.5 UltrAufoil TEM grid could allow for data collection rates of over 17 000 micrographs per day using a Talos Arctica and Gatan K3 DED.

## 4. Discussion

Here, we highlight the performance of the 200 keV Talos Arctica equipped with a Gatan K3 DED under various imaging conditions using *SerialEM* automated data collection software. We performed analysis at multiple image shift delay factor values in both super-resolution and hardware-binning mode and observed no significant difference in data quality between datasets, generating a 1.8 Å structure of apoferritin from as little as 56 min of data collection. Implementation of this data collection method allows for acquisition of over 12 000 movies per day, with the potential to increase data collection rates further by collecting more images per stage position. This can be accomplished by increasing the BIS distance, acquiring multiple shots per hole on a 300 keV instrument or using custom hole spacing.

### 4.1. Comparison of super-resolution mode and hardware-binning mode

The advantages of collecting data in super-resolution have been shown to be most beneficial at lower magnifications, achieving resolutions beyond physical Nyquist for symmetric proteins at pixel sizes of 1.66 and 2.1 Å (Feathers *et al.*, 2021 ▸; Sun *et al.*, 2021 ▸). Taking advantage of the larger field of view, we expect faster data collection as measured by counting the number of particles per hour when operating the DED in super-resolution mode, by a factor of ∼2 for magnifications with equivalent super-resolution and hardware-binned pixels sizes. With this microscope setup and a calibrated physical pixel size of 0.88 Å, we found no significant differences in EM map resolutions for apoferritin between hardware-binned mode (5760 × 4092 pixels) and super-resolution mode (11520 × 8184 pixels). Interestingly, we found data collection speeds of hardware-binned mode to be 52% faster than data collected in super-resolution mode. The lack of appreciable difference under these imaging conditions may be due to the use of reduction by antialias filtering instead of strict binning in *SerialEM*, which allows hardware-binned data to retain some benefits of collection in super-resolution mode. Microscope setup, detector pixel size, variation in image shift delay tolerances, particle density, hole size and spacing, target resolution, and desired data collection speed all need to be considered when deciding to utilize super-resolution mode or hardware-binning during automated data collection with a Gatan K3 DED.

### 4.2. Collecting high-resolution cryo-EM data at a rate of over 12 000 movies per day with a Talos Arctica G3 and Gatan K3 DED

In this study, we show it is possible to obtain a 1.8 Å EM map with a 200 keV Talos Arctica cryo-TEM equipped with a Gatan K3 DED at a data collection rate of 524 movies per hour. We attribute the increased speeds of data collection to the following factors: (1) using UltrAufoil R0.6/1 grids, (2) collection using BIS up to ∼7 µm, (3) lowering the image shift delay factor to 0 and (4) hardware-binning. Using this setup, data quality did not appear compromised by large BIS distances or by reducing the image shift delay factor, as the EM maps had similar features and resolutions (1.8–1.9 Å). To further explore the impact of BIS distance and reducing image shift delay on data quality, we collected 5881 movies from a single UltrAufoil grid of mouse apoferritin and obtained an EM map at 1.78 Å resolution. The final EM maps reconstructed from particles grouped by multishot position showed no significant difference in resolution and EM map quality. Taken together, this study clearly shows that it is possible to routinely collect high-resolution cryo-EM data using Talos Arctica at a rate of over 12 000 movies per day.

### 4.3. How to acquire 700+ movies per hour using 200 keV cryo-TEM

After reducing the image shift delay factor to 0 and observing no measurable difference in data quality, we sought to explore other limitations on data collection speed. Other research has shown it feasible to use a total dose of ∼7 e^−^ Å^−2^ and obtain a 3 Å reconstruction of apoferritin (Bepler *et al.*, 2020 ▸). By setting the total dose to ∼16 e^−^ Å^−2^, we can reduce our exposure time to 0.9 s. With the image shift delay set to 0, the other *SerialEM* delays that are necessary for high-quality data collection (shutter delay, *etc*.) sum to approximately 1.3 s of delay before each exposure. With these settings, we measured the total time to collect a single movie to be approximately 4.5 s. If we disregard the time required for stage movement, autofocus and drift measurement and only consider the previously mentioned delay time, exposure time, camera read out and write time, the maximum speed with this setup is 800 movies per hour. Even on a 300 keV microscope collecting multiple shots per hole and requiring fewer stage movements, the total time required for each movie collected would need to be reduced to less than 3.6 s to reach 1000 movies per hour. The smallest allowable beam diameter for the Talos Arctica used in this study is 1.6 µm, making the ideal hole spacing R0.6/0.5. This provides sufficient spacing to prevent double exposure of each movie target area while maximizing the number of movies collected per stage position. Applying this spacing on a lacey carbon grid enabled us to obtain a data collection rate of 720 movies per hour using a 9 × 9 multishot array with a maximum BIS of ∼6.2 µm. The lower resolution of the EM map acquired from this data can likely be attributed to particles picked from the lacey carbon being incorporated into the reconstruction and greater electron-induced specimen movement on lacey carbon compared with ultrastable gold films (Russo & Passmore, 2014 ▸). We do not believe that the image shift delay or BIS distance had a negative impact on data quality, as similar parameters were used on other data collections and produced 1.8 Å reconstructions. The development of a cryogrid with R0.6/0.5 spacing will allow for further exploration of optimum imaging conditions using this microscope and camera setup.

### 4.4. Utility of 200 keV cryo-TEM for high-speed, high-resolution cryo-EM data collection

In the past few years, coinciding with advancements in microscope hardware and software technology, the field of cryo-EM has been growing steadily and is now utilized by a wide range of researchers (Nogales, 2016 ▸). Access to cryo-TEMs capable of screening cryo-grids as well as microscopes that are capable of high-resolution data collection is often a bottleneck in the process of structural determination. Several national centers have been established to provide individual researchers access to such microscopes, as well as training in cryo-TEM (Eng *et al.*, 2019 ▸). However, as more individuals begin to utilize cryo-EM as a structural biology technique, the demand for microscope beam time for both screening and data collection will increase. It follows that the need to develop procedures for increasing data collection speeds is becoming more important. As data acquisition speeds increase, so will the cost savings associated with beam time, especially for cryo-TEMs equipped with DEDs. It is often necessary to collect data to determine if a sample is amenable to high-resolution structural determination (Passmore & Russo, 2016 ▸), or to define imaging conditions on the fly (Gómez-Blanco *et al.*, 2018 ▸; Thompson *et al.*, 2019 ▸). Having protocols to collect movies at rate of ≥500 per hour is critical in expanding the capabilities of 200 keV cryo-TEMs to obtain multiple structures from a single screening session (5–8 h). For heterogenous protein complexes, collecting larger datasets of 30 000–50 000 movies is becoming more common and requires access to microscopes capable of fast data collection without sacrificing data quality (Naydenova *et al.*, 2021 ▸; Seven *et al.*, 2021 ▸). We show that it is possible to obtain high-resolution data collected on a 200 keV Talos Arctica G3 cryo-TEM using *SerialEM* by BIS, at speeds of up to 520 movies per hour for an R0.6/1 grid. Moreover, we demonstrate even faster rates of data acquisition are possible (720 movies per hour with lower exposure times). Data collection speeds were dependent on binning, hole size and spacing, and image shift delay factor set in *SerialEM*. Hardware limitations of data conversion and transfer are likely the next frontiers in the race for faster data acquisition speeds. We assert that the utility of this procedure can be extended to other 200 and 300 keV cryo-TEMs to maximize beam-time efficiency by increasing data collection speeds without compromising data quality and enabling high-throughput, high-resolution data collection.

## 5. Data availability

The cryo-EM maps of mouse heavy chain apoferritin have been deposited in the EMDB with the following accession codes: hardware-binned overnight (ISDF 0.5) 1.78 Å, EMD-25839; hardware-binned lacey (ISDF 0) 2.19 Å, EMD-25840;  hardware-binned (ISDF 0) 1.83 Å, EMD-25841; super-resolution (ISDF 0) 1.85 Å, EMD-25842.

## Supplementary Material

Supporting figures and tables. DOI: 10.1107/S2052252522000069/eh5014sup1.pdf

EMDB reference: mouse heavy-chain apoferritin, 1.78 Å, EMD-25839

EMDB reference: 2.19 Å, EMD-25840

EMDB reference: 1.83 Å, EMD-25841

EMDB reference: 1.85 Å, EMD-25842

## Figures and Tables

**Table 1:** Data collection and EM map statistics

Data collection	Super-resolution	Hardware-binned	Lacey hardware-binned	Hardware-binned overnight
Microscope	Talos Arctica G3	Talos Arctica G3	Talos Arctica G3	Talos Arctica G3
Voltage (keV)	200	200	200	200
Nominal magnification	54900	54900	54900	88400
Detector	Gatan K3	Gatan K3	Gatan K3	Gatan K3
Pixel size (Å)	0.44	0.88	0.88	0.545
Multishot	7 × 7	7 × 7	9 × 9	7 × 7
Image shift delay factor	0, 0.25, 0.5, 1.0, 2.0	0, 0.5, 1.0	0	0.5
Cumulative exposure (e^−^ Å^−2^)	45.367	45.367	16.2	54.3
Exposure rate (e^−^ pixel^−1^ s^−1^)	12.9	12.9	13.8	11.2
Exposure per frame (e^−^ Å^−2^)	0.756	0.756	0.81	1.438
Defocus range (µm)	0.46–1.59	0.46–1.49	0.46–1.86	0.20–1.80
Movies collected	490	490	1134	5881
Data collection rate (movies per hour)	346, 348, 342, 296, 240	524, 488, 426	720	500
				
EM Map				
Final particles (No.)	313040	313040	219350	1111931
Symmetry imposed	O	O	O	O
Resolution at FSC 0.143 Å (unmasked/masked)	2.1/1.85, 2.0/1.83, 2.0/1.81, 2.0/1.81, 2.0/1.80	2.0/1.83, 2.1/1.90 2.0/1.81	2.54/2.19	1.95/1.78
Map-sharpening *B*-factor (Å)	60.2, 56.8, 55.2, 55.7, 55	57.4, 68.9, 55.9	76.5	57.2
Fraction of physical Nyquist	0.95, 0.96, 0.97, 0.97, 0.98	0.96, 0.93, 0.97	0.80	0.61

**Table 2:** Subgroup analysis of overnight data collection

Image shift (µm)	Number of holes	Image shift delay (s)†	Number of particles in each group	Number of particles in EM map	Resolution (Å)	*B*-factor
6.79	3	0.8	66386	20564	2.06	41
6.79	1	3.4	20564	20564	2.06	42.5
6.79	4	3.4, 0.8	86950	23445	2.06	43.4
5.77	8	0.8	176130	23445	2.02	41.5
5.06	8	0.8	181108	23445	2.04	42.6
4.8	4	0.8	90289	23445	2.04	43.1
4.53	4	0.8	90378	23445	2.03	41.7
3.58	8	0.8	185819	23445	2.02	42
3.2	4	0.8	93618	23445	2.03	42.5
2.26	4	0.8	92144	23445	2.03	42.2
1.6	4	0.8	92050	23445	2.06	42.2
0	1	0.8	23445	23445	2.08	43

† Based on output from the *SerialEM* properties file.

## References

[bb1] Bepler, T., Kelley, K., Noble, A. J. & Berger, B. (2020). *Nat. Commun.* **11**, 5208. 10.1038/s41467-020-18952-1 PMC7567117 33060581

[bb2] Bromberg, R., Guo, Y., Borek, D. & Otwinowski, Z. (2020). *IUCrJ*, **7**, 445–452. 10.1107/S2052252520002444 PMC7201289 32431828

[bb3] Campbell, M. G., Kearney, B. M., Cheng, A., Potter, C. S., Johnson, J. E., Carragher, B. & Veesler, D. (2014). *J. Struct. Biol.* **188**, 183–187. 10.1016/j.jsb.2014.09.008 PMC4497823 25278130

[bb4] Cao, C., Kang, H. J., Singh, I., Chen, H., Zhang, C., Ye, W., Hayes, B. W., Liu, J., Gumpper, R. H., Bender, B. J., Slocum, S. T., Krumm, B. E., Lansu, K., McCorvy, J. D., Kroeze, W. K., English, J. G., DiBerto, J. F., Olsen, R. H. J., Huang, X.-P., Zhang, S., Liu, Y., Kim, K., Karpiak, J., Jan, L. Y., Abraham, S. N., Jin, J., Shoichet, B. K., Fay, J. F. & Roth, B. L. (2021). *Nature*, **600**, 170–175. 10.1038/s41586-021-04126-6 PMC9150435 34789874

[bb5] Cardone, G., Heymann, J. B. & Steven, A. C. (2013). *J. Struct. Biol.* **184**, 226–236. 10.1016/j.jsb.2013.08.002 PMC3837392 23954653

[bb6] Cash, J. N., Kearns, S., Li, Y. & Cianfrocco, M. A. (2020). *IUCrJ*, **7**, 1179–1187. 10.1107/S2052252520013482 PMC7642776 33209328

[bb7] Cheng, A., Eng, E. T., Alink, L., Rice, W. J., Jordan, K. D., Kim, L. Y., Potter, C. S. & Carragher, B. (2018). *J. Struct. Biol.* **204**, 270–275. 10.1016/j.jsb.2018.07.015 PMC6163078 30055234

[bb8] Cheng, A., Negro, C., Bruhn, J. F., Rice, W. J., Dallakyan, S., Eng, E. T., Waterman, D. G., Potter, C. S. & Carragher, B. (2021). *Protein Sci.* **30**, 136–150. 10.1002/pro.3967 PMC7737759 33030237

[bb9] Cheng, Y. (2015). *Cell*, **161**, 450–457. 10.1016/j.cell.2015.03.049 PMC4409662 25910205

[bb10] Chiu, P. L., Li, X., Li, Z., Beckett, B., Brilot, A. F., Grigorieff, N., Agard, D. A., Cheng, Y. & Walz, T. (2015). *J. Struct. Biol.* **192**, 163–173. 10.1016/j.jsb.2015.08.015 PMC4633381 26318383

[bb11] Danev, R., Belousoff, M., Liang, Y. L., Zhang, X., Eisenstein, F., Wootten, D. & Sexton, P. M. (2021). *Nat. Commun.* **12**, 4333. 10.1038/s41467-021-24650-3 PMC8282782 34267200

[bb12] Eng, E. T., Rice, W. J., Cheng, A., Carragher, B. & Potter C. S. (2019). *Microsc. Microanal.* **25**, 2.

[bb13] Efremov, R. G. & Stroobants, A. (2021). *Acta Cryst.* D**77**, 555–564. 10.1107/S2059798321002151 PMC8098478 33950012

[bb14] Feathers, J. R., Spoth, K. A. & Fromme, J. C. (2021). *J. Struct. Biol. X*, **5**, 100047. 10.1016/j.yjsbx.2021.100047 PMC8008246 33817625

[bb15] Glaeser, R. M., Typke, D., Tiemeijer, P. C., Pulokas, J. & Cheng, A. (2011). *J. Struct. Biol.* **174**, 1–10. 10.1016/j.jsb.2010.12.005 PMC3056894 21182964

[bb16] Gómez-Blanco, J., de la Rosa-Trevín, J. M., Marabini, R., del Cano, L., Jiménez, A., Martínez, M., Melero, R., Majtner, T., Maluenda, D., Mota, J., Rancel, Y., Ramírez-Aportela, E., Vilas, J. L., Carroni, M., Fleischmann, S., Lindahl, E., Ashton, A. W., Basham, M., Clare, D. K., Savage, K., Siebert, C. A., Sharov, G. G., Sorzano, C. O. S., Conesa, P. & Carazo, J. M. (2018). *J. Struct. Biol.* **204**, 457–463. 10.1016/j.jsb.2018.10.001 PMC6303188 30296492

[bb17] Grimm, R., Singh, H., Rachel, R., Typke, D., Zillig, W. & Baumeister, W. (1998). *Biophys. J.* **74**, 1031–1042. 10.1016/S0006-3495(98)74028-7 PMC1302584 9533716

[bb18] Guo, H., Franken, E., Deng, Y., Benlekbir, S., Singla Lezcano, G., Janssen, B., Yu, L., Ripstein, Z. A., Tan, Y. Z. & Rubinstein, J. L. (2020). *IUCrJ*, **7**, 860–869. 10.1107/S205225252000929X PMC7467176 32939278

[bb19] Hamdi, F., Tüting, C., Semchonok, D. A., Visscher, K. M., Kyrilis, F. L., Meister, A., Skalidis, I., Schmidt, L., Parthier, C., Stubbs, M. T. & Kastritis, P. L. (2020). *PLoS One*, **15**, e0232540. 10.1371/journal.pone.0232540 PMC7202636 32374767

[bb20] Herzik, M. A., Wu, M. & Lander, G. C. (2019). *Nat. Commun.* **10**, 1032. 10.1038/s41467-019-08991-8 PMC6399227 30833564

[bb21] Herzik, M. A. Jr (2021). *Methods Mol. Biol.* **2215**, 125–144. 10.1007/978-1-0716-0966-8_6 33368002

[bb22] Herzik, M. A. Jr, Wu, M. & Lander, G. C. (2017). *Nat. Methods*, **14**, 1075–1078. 10.1038/nmeth.4461 PMC5679434 28991891

[bb23] Kim, L. Y., Rice, W. J., Eng, E. T., Kopylov, M., Cheng, A., Raczkowski, A. M., Jordan, K. D., Bobe, D., Potter, C. S. & Carragher, B. (2018). *Front. Mol. Biosci.* **5**, 50. 10.3389/fmolb.2018.00050 PMC6009202 29951483

[bb24] Li, X., Zheng, S. Q., Egami, K., Agard, D. A. & Cheng, Y. (2013). *J. Struct. Biol.* **184**, 251–260. 10.1016/j.jsb.2013.08.005 PMC3854003 23968652

[bb25] Mastronarde, D. N. (2005). *J. Struct. Biol.* **152**, 36–51. 10.1016/j.jsb.2005.07.007 16182563

[bb26] Mastronarde, D. N. (2021). *SerialEM*, https://bio3d.colorado.edu/SerialEM/hlp/html/menu_calibration.html.

[bb27] Mendez, J. H., Mehrani, A., Randolph, P. & Stagg, S. (2019). *IUCrJ*, **6**, 1007–1013. 10.1107/S2052252519012661 PMC6830211 31709056

[bb28] Merk, A., Fukumura, T., Zhu, X., Darling, J. E., Grisshammer, R., Ognjenovic, J. & Subramaniam, S. (2020). *IUCrJ*, **7**, 639–643. 10.1107/S2052252520006855 PMC7340270 32695410

[bb29] Mills, D. J. (2021). *Q. Rev. Biophys.* **54**, e2. 10.1017/S003358352000013X 33413714

[bb30] Nakane, T., Kotecha, A., Sente, A., McMullan, G., Masiulis, S., Brown, P., Grigoras, I. T., Malinauskaite, L., Malinauskas, T., Miehling, J., Uchański, T., Yu, L., Karia, D., Pechnikova, E. V., de Jong, E., Keizer, J., Bischoff, M., McCormack, J., Tiemeijer, P., Hardwick, S. W., Chirgadze, D. Y., Murshudov, G., Aricescu, A. R. & Scheres, S. H. W. (2020). *Nature*, **587**, 152–156. 10.1038/s41586-020-2829-0 PMC7611073 33087931

[bb31] Naydenova, K., Muir, K. W., Wu, L. F., Zhang, Z., Coscia, F., Peet, M. J., Castro-Hartmann, P., Qian, P., Sader, K., Dent, K., Kimanius, D., Sutherland, J. D., Lowe, J., Barford, D. & Russo, C. J. (2021). *Proc. Natl Acad. Sci. USA*, **118**, e2021946118. 10.1073/pnas.2021946118 PMC7896311 33526596

[bb32] Nogales, E. (2016). *Nat. Methods*, **13**, 24–27. 10.1038/nmeth.3694 PMC4913480 27110629

[bb33] Passmore, L. A. & Russo, C. J. (2016). *Methods Enzymol.* **579**, 51–86. 10.1016/bs.mie.2016.04.011 PMC5140023 27572723

[bb34] Patwardhan, A. (2017). *Acta Cryst.* D**73**, 503–508. 10.1107/S2059798317004181 PMC5458492 28580912

[bb35] Pettersen, E. F., Goddard, T. D., Huang, C. C., Couch, G. S., Greenblatt, D. M., Meng, E. C. & Ferrin, T. E. (2004). *J. Comput. Chem.* **25**, 1605–1612. 10.1002/jcc.20084 15264254

[bb36] Punjani, A., Rubinstein, J. L., Fleet, D. J. & Brubaker, M. A. (2017). *Nat. Methods*, **14**, 290–296. 10.1038/nmeth.4169 28165473

[bb37] Punjani, A., Zhang, H. & Fleet, D. J. (2020). *Nat. Methods*, **17**, 1214–1221. 10.1038/s41592-020-00990-8 33257830

[bb38] Russo, C. J. & Passmore, L. A. (2014). *Science*, **346**, 1377–1380. 10.1126/science.1259530 PMC4296556 25504723

[bb39] Sader, K., Matadeen, R., Castro Hartmann, P., Halsan, T. & Schlichten, C. (2020). *Acta Cryst.* D**76**, 313–325. 10.1107/S2059798320002223 PMC7137108 32254055

[bb40] Schorb, M., Haberbosch, I., Hagen, W. J. H., Schwab, Y. & Mastronarde, D. N. (2019). *Nat. Methods*, **16**, 471–477. 10.1038/s41592-019-0396-9 PMC7000238 31086343

[bb41] Seven, A. B., Barros-Álvarez, X., de Lapeyrière, M., Papasergi-Scott, M. M., Robertson, M. J., Zhang, C., Nwokonko, R. M., Gao, Y., Meyerowitz, J. G., Rocher, J. P., Schelshorn, D., Kobilka, B. K., Mathiesen, J. M. & Skiniotis, G. (2021). *Nature*, **595**, 450–454. 10.1038/s41586-021-03680-3 PMC8822903 34194039

[bb42] Song, B., Lenhart, J., Flegler, V. J., Makbul, C., Rasmussen, T. & Böttcher, B. (2019). *Ultramicroscopy*, **203**, 145–154. 10.1016/j.ultramic.2019.01.002 30738626

[bb43] Sun, M., Azumaya, C. M., Tse, E., Bulkley, D. P., Harrington, M. B., Gilbert, G., Frost, A., Southworth, D., Verba, K. A., Cheng, Y. & Agard, D. A. (2021). *J. Struct. Biol.* **213**, 107745. 10.1016/j.jsb.2021.107745 33984504

[bb44] Thompson, R. F., Iadanza, M. G., Hesketh, E. L., Rawson, S. & Ranson, N. A. (2019). *Nat. Protoc.* **14**, 100–118. 10.1038/s41596-018-0084-8 PMC7618007 30487656

[bb45] Weis, F. & Hagen, W. J. H. (2020). *Acta Cryst.* D**76**, 724–728. 10.1107/S2059798320008347 PMC7397495 32744254

[bb46] Wu, C., Huang, X., Cheng, J., Zhu, D. & Zhang, X. (2019). *J. Struct. Biol.* **208**, 107396. 10.1016/j.jsb.2019.09.013 31562921

[bb47] Wu, M., Lander, G. C. & Herzik, M. A. Jr (2020). *J. Struct. Biol. X*, **4**, 100020. 10.1016/j.yjsbx.2020.100020 PMC7337053 32647824

[bb48] Xie, Q., Yoshioka, C. K. & Chapman, M. S. (2020). *Viruses*, **12**, 1194. 10.3390/v12101194 PMC7589773 33092282

[bb49] Yip, K. M., Fischer, N., Paknia, E., Chari, A. & Stark, H. (2020). *Nature*, **587**, 157–161. 10.1038/s41586-020-2833-4 33087927

[bb50] Zemlin, F. W. K., Weiss, K., Schiske, P., Kunath, W. & Herrmann, K. (1978). *Ultramicroscopy*, **3**, 49–60.

[bb51] Zhang, X. & Hong Zhou, Z. (2011). *J. Struct. Biol.* **175**, 253–263. 10.1016/j.jsb.2011.05.004 PMC3710782 21627992

[bb52] Zhu, D., Wang, X., Fang, Q., Van Etten, J. L., Rossmann, M. G., Rao, Z. & Zhang, X. (2018). *Nat. Commun.* **9**, 1552. 10.1038/s41467-018-04051-9 PMC5908801 29674632

[bb53] Zivanov, J., Nakane, T., Forsberg, B. O., Kimanius, D., Hagen, W. J., Lindahl, E. & Scheres, S. H. (2018). *eLife* **7**, e42166. 10.7554/eLife.42166 PMC6250425 30412051

