# Supplementary material for: High-speed high-resolution data collection on a 200 keV cryo-TEM
Source: IUCrJ. 2022 Jan 29;9(Pt 2):243–52. doi: 10.1107/S2052252522000069 (PMC8895008; doi:10.1107/S2052252522000069)
Supplement: Supplementary file 1 [file m-09-00243-sup1.pdf]

IUCrJ

Volume 9 (2022)

Supporting information for article:

High-speed High-resolution Data Collection on a 200 keV Cryo-TEM

Jared V. Peck, Jonathan F. Fay and Joshua D. Strauss

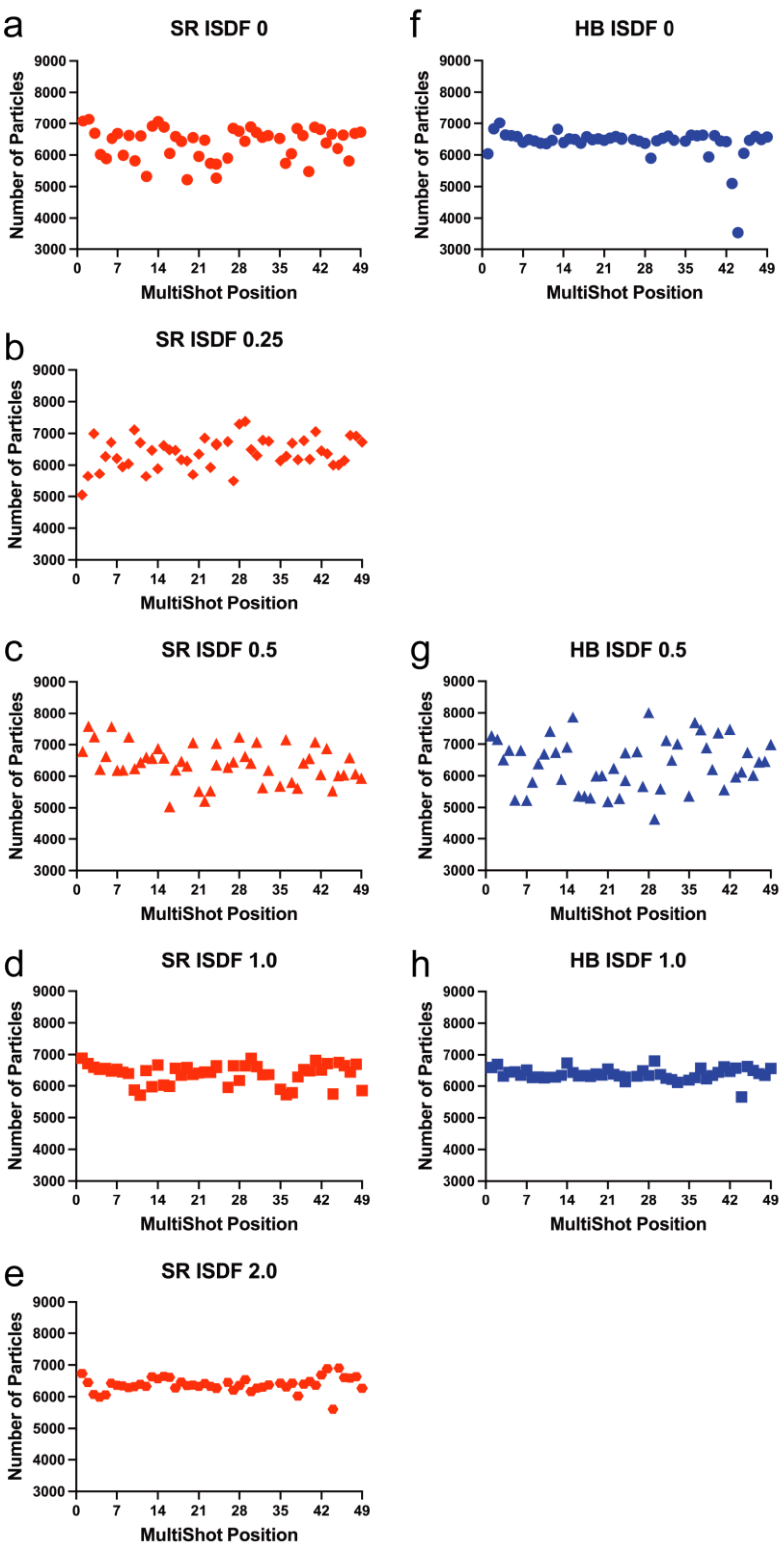

Figure S1. Number of Particles by Multishot Position for Data Collection with Different Image Shift Delay Factors First-level heading. (*a-h*) Number of particles contributed to final EM map according to order taken within the multishot array. Dataset collected in super-resolution (SR, indicated in red) or hardware binned (HB, indicated in blue). Image shift delay factor (ISDF) was set to either 0, 0.25, 0.5, 1 or 2.

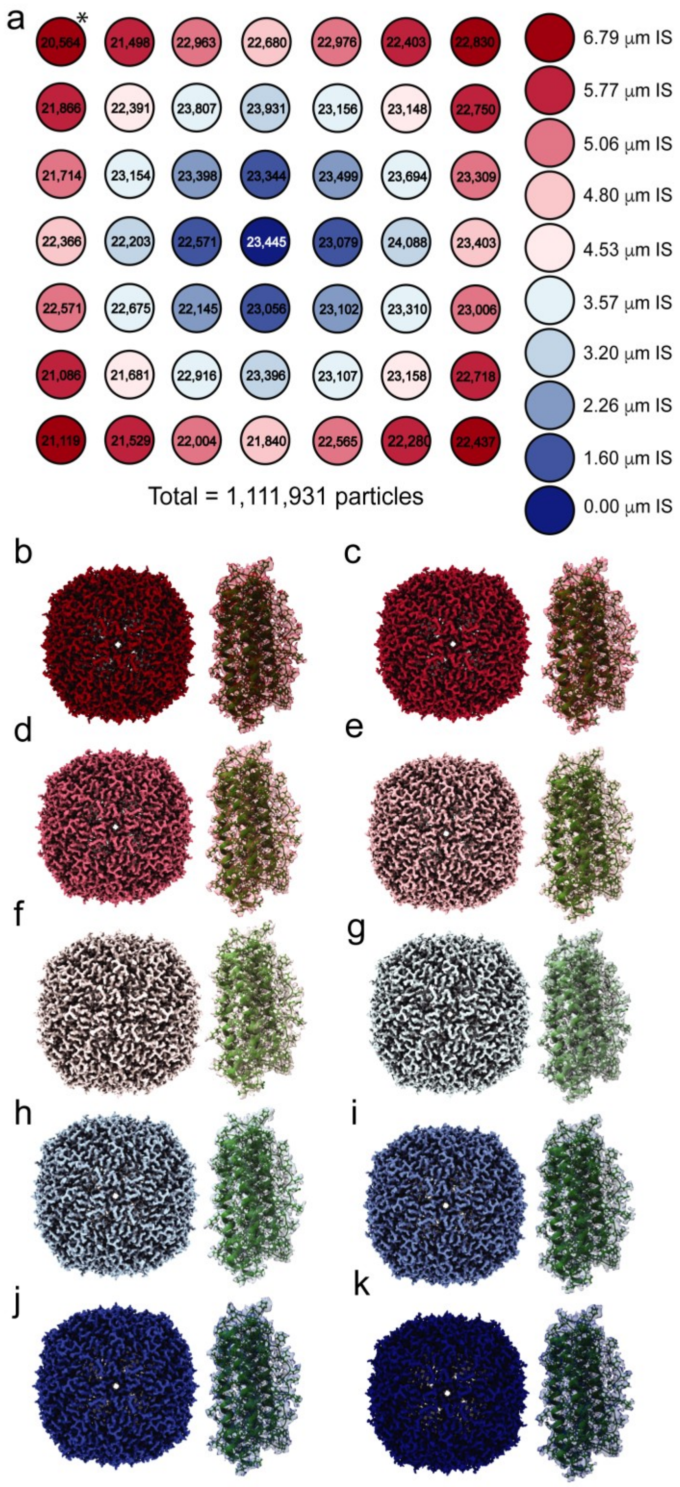

Figure S2. Particle Contribution to Final EM Map by Multishot Position. Particles (1,111,931) included in the final 1.78 Å EM map were separated into 10 groups based on image shift (IS) distance and processed independently. (*a*) The number of particles in each of the 7x7 multishot positions are indicated in the coloured circles. Position 1, marked here with an asterisk, was the only position that showed a significant difference, contributing 10% fewer particles than average. (*b*-*k*) EM maps reconstructed from particles grouped together based on image shift distance. The EM maps showed no significant difference in resolution or B-Factor.

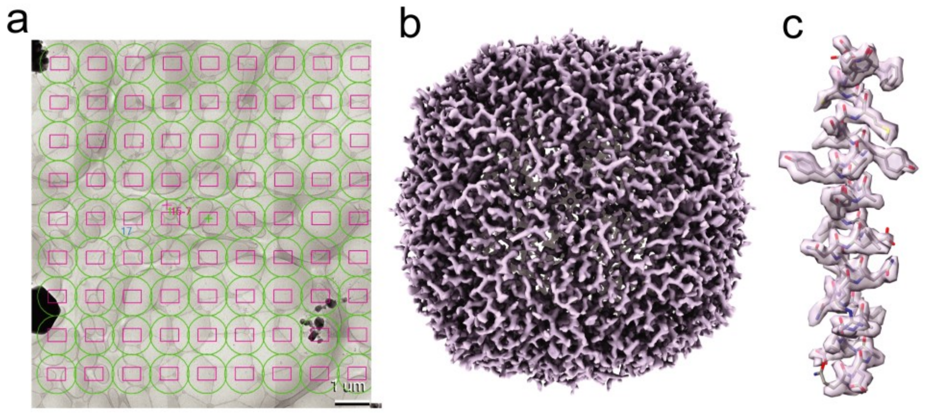

Figure S3. High-speed Data Collection on a Lacey TEM grid. (*a*) A single 9x9 multishot array on a Lacey carbon TEM grid. Green circles indicate beam diameter, the position of the Gatan K3 detector in pink. (*b*) Isosurface rendering of mouse apoferritin collected on a single grid square at 720 movies per hour. (*c*) Atomic model of mouse apoferritin PDB 6v21 docked into EM map, closeup of helix 1 GLN14 to ASP42.

Table S1

Electron Beam Diameter

**50 µm Condenser Aperture**				
**Spot Size**	**% C2 Parallel Illumination**	**Beam Diameter (µm)**	**Flux (e^-/pixel/sec)**	**Dose Rate (e-/$Å^2$/sec)**
1	46.461	1.73	29.94	36.16
2	44.829	1.599	25.54	30.84
3	43.2	1.67	15.58	18.81
4	41.891	1.632	10.47	12.64
5	40.826	1.695	6.16	7.44
6	39.288	1.567	3.84	4.64
7	38.468	1.64	1.82	2.2
8	37.952	1.62	0.6	0.72
**Average Beam Diameter (µm)**		**1.64**		

* Measurements made at nominal magnification of 54,900 X at the detector level, corresponding to pixel size of 0.88 Å. The objective and selected area aperture were removed. Gun lens was set to 4.
